# Effects of Concentrate Levels in Prepartum Diet on Milk Performance, Energy Balance and Rumen Fermentation of Transition Montbéliarde–Holstein Crossbred Cows

**DOI:** 10.3390/ani12091051

**Published:** 2022-04-19

**Authors:** Zhantao Yang, Shuangzhao Dong, Yuhui Zheng, Fanlin Kong, Jiaying Lv, Xiaoge Sun, Yajing Wang, Zhijun Cao, Wei Wang, Shengli Li

**Affiliations:** State Key Laboratory of Animal Nutrition, College of Animal Science and Technology, China Agricultural University, Beijing 100193, China; yzt980929@163.com (Z.Y.); zhaoddr@163.com (S.D.); zhengyuhui@cau.edu.cn (Y.Z.); a895833622@163.com (F.K.); lvjiaying1123@163.com (J.L.); xiaogesun@163.com (X.S.); yajingwang@cau.edu.cn (Y.W.); caozhijun@cau.edu.cn (Z.C.)

**Keywords:** Montbéliarde–Holstein crossbred cows, concentrate levels, energy balance, production performance, rumen fermentation

## Abstract

**Simple Summary:**

The transition period (3 wks before to 3 wks after calving) is an important period in the lactation cycle of dairy cows. During this period, dairy cows undergo a series of physiological and metabolic changes due to the demands of pregnancy, parturition and postpartum lactation, which can easily lead to a negative energy balance (NEB). Therefore, the energy balance and nutritional regulation mechanism of dairy cows during the transition period are the focus of dairy cow nutrition and physiology research. However, crossbred cows have received less attention to date. Our study showed that feeding a prenatal concentrate mix at a rate of 0.6% of the body weight of Montbéliarde–Holstein crossbred cows has no negative effect on the performance and rumen fermentation of postpartum dairy cows and can satisfy the energy needs of dairy cows in the prepartum period. Furthermore, our data provide a theoretical basis for further revisions to the feeding standard and to alleviate the NEB of Montbéliarde–Holstein crossbred cows in China.

**Abstract:**

This study was conducted to investigate the effect of three rates of prepartum dietary concentrate feeding on the milk performance, energy balance, and rumen fermentation of Montbéliarde–Holstein crossbred cows. Eighteen transition Montbéliarde–Holstein crossbred cows with similar days of gestation (258 ± 12 day) and body weights (622 ± 44 kg) were selected and randomly divided into three groups. In the prepartum period, the addition of concentrates accounted for 0.3% (low concentrate, LC), 0.6% (medium concentrate, MC), and 0.9% (high concentrate, HC) of the cow’s body weight. The forage was corn stover, which was fed to the cows ad libitum with free access to water. Postpartum, all of the cows were fed a common lactation total mixed ration. The experimental period lasted from 21 days prepartum to 28 days postpartum. The energy balance (EB), net energy intake (NEI), and dry matter intake (DMI) of the HC group were greater than those of the other groups (*p* < 0.05). Likewise, the non-esterified fatty acids (NEFA), β-hydroxybutyric acid (BHBA), and total bilirubin (TBIL) in the blood of the LC group had significantly higher concentrations than they did in the other groups (*p* < 0.05). Moreover, the increase in the level of dietary concentrate had no significant effect on the rumen fermentation parameters (*p* > 0.05), and the total intestinal digestibility of dry matter (DM), crude protein (CP), and ether extract (EE) in the HC group was significantly higher (*p* < 0.05) than it was in the other groups during the prepartum period. In conclusion, the administration of the MC diet in the prepartum period had no negative effect on the performance and rumen fermentation of postpartum dairy cows and can satisfy the energy needs of prepartum dairy cows. Therefore, under our experimental conditions, the 0.6% prenatal concentrate feeding amount was the most appropriate for Montbéliarde–Holstein crossbred cows.

## 1. Introduction

In dairy cows, the transition period is usually defined as the three weeks before calving and the three weeks after calving [1,2]. During this period, dairy cows experience calving stress, changes in their diet structure, and so on, which cause huge physiological and metabolic challenges that can affect their health and well-being either directly or indirectly. Ensuring consistent feed intake during this period can alleviate the cow from entering a state of negative energy balance (NEB), thereby reducing the risk of metabolic diseases, such as ketosis [3]. However, research has shown that the dry matter intake (DMI) of dairy cows during the transition period drops by about 30% [4]. Additionally, it is difficult or impossible to meet the increased nutrient demands of cattle due to restricted feed intake, which results in the mobilization of the fat stores as well as in the muscle tissue of cows to compensate for the deficits in dietary nutrients [5]. This imbalance between supply and demand leads to NEB in dairy cows, contributing to the high incidence of metabolic disease during this period [6,7]. The clinical symptoms of NEB mainly include an increase in body fat breakdown and a decrease in insulin sensitivity, and excessive body fat breakdown can lead to fatty liver and ketosis, among other conditions [8].

Previous studies pointed out that high-energy diets should be fed before delivery to maximize prenatal DMI and are aimed to promote pregnancy and mammary gland development, preparing cows for a better adaptation to early lactation diets [9,10]. However, subsequent research demonstrated that prenatal overfeeding caused increased body fat mobilization in dairy cows, something that could lead to more serious NEB and cause metabolic disorders in dairy cows after delivery [11,12]. In recent years, studies on the energy levels of Holstein cows in the perinatal period have shown that feeding a high-energy density diet to dairy cows during the prepartum period impairs metabolic status in early lactation [13] and that feeding a low-energy diet to dairy cows in the prepartum period is more conducive to controlling the prenatal net energy intake (NEI), increasing the milk production and DMI of postpartum cows, and reducing an NEB postpartum [14]. However, there is little research on the energy requirements of crossbred Simmental and Holstein cows.

Therefore, this experiment aims to investigate the effect of prepartum dietary concentrate levels on the milk performance, energy balance, and rumen fermentation of Montbéliarde–Holstein crossbred cows to provide a theoretical basis for further revisions to the feeding standards and to alleviate the NEB of Montbéliarde–Holstein crossbred cows in China. In this study, we hypothesized that providing Montbéliarde–Holstein crossbred cows in the transition period with more concentrate would increase NEI, thereby alleviating problems related to NEB problems, reduce the NEFA and BHBA concentrations in serum, and increase milk production during the postpartum period.

## 2. Materials and Methods

### 2.1. Experiment Animals

Eighteen perinatal cows were selected from Hengli Dairy Farm (Anshan, China). All experimental procedures were approved by the Ethical Committee of the College of Animal Science and Technology of China Agriculture University (Protocol number: 2020-5-LZ).

### 2.2. Experiment Materials and Design

Eighteen Montbéliarde–Holstein crossbred cows who were in the transition period and who had similar gestation days (258 ± 12 day) and body weights (622 ± 44 kg) were selected and randomly divided into three groups. In the prepartum period, the experimental cows were fed three times per day (07:00, 12:00, 17:00) in a single trough, and the forage first and then concentrate feeding method was adopted; the forage was corn stover, which was fed ad libitum with free access to water. According to research of Huang et al. and the NRC (2001) [14,15], the added concentrations accounted for 0.3% (low concentrate, LC), 0.6% (medium concentrate, MC), and 0.9% (high concentrate, HC) of the cows’ body weight. At 7 d before expected parturition, the cows were moved to individual maternity pens until parturition. After calving, the three groups were fed a common lactation TMR three times a day at 07:00, 12:00, and 17:00 and had free access to water. The cows were milked twice daily at 06:30 and 18:30, and the milk yield was recorded from one week postpartum. The pre-feeding period began from 25 days prepartum, and the experiment period lasted from 21 days prepartum to 28 days postpartum. The diet for the pre-feeding period was the same as the prepartum diet during the experimental period. The dietary composition (dry matter basis) and nutrient composition are shown in Table 1 and Table 2.

### 2.3. Sample Collection and Analysis

#### 2.3.1. Collection of Body Weight, Body Condition Score and Energy Balance Data

The body weight (BW) of each cow was measured every seven days at 06:30. The net energy intake (NEI) was determined by multiplying the weekly DMI by the calculated energy value of the diet. The energy required for body maintenance (NEM) was calculated using the equation NEM = BW^0.75^ × 0.08. Pregnancy requirements (NEP) were computed using the equation NEP = [(0.00318 × days of gestation − 0.0353) × (calf birth weight/45)]/0.218. Milk energy was calculated using the equation NEL = MP × [(0.0929 × Fat) + (0.0547 × Prot) + (0.0395 × Lact)], where MP, Fat, Prot, and Lact are the milk production (kg), fat, CP, and lactose percentages in milk, respectively [16]. The estimated energy balance (EB) prepartum was calculated using the equation EB = NEI − (NEM + NEP), and the estimated energy balance postpartum was computed using the equation EB = NEI − (NEM + NEL). The body condition score (BCS) was determined by the internationally accepted 1–5 scale standard [17], and experienced people were assessed independently.

#### 2.3.2. Collection and Analysis of Dry Diets and Fecal Samples

The DMI was calculated by the daily feed offered and orts, and samples of the diets were collected every seven days. About 300 g of fecal samples was collected on the 8th, 7th, and 6th days prepartum and on the 13th, 14th, and 15th days postpartum by means of rectal fecal collection. Equal pre- and postpartum fecal samples were taken from each cow. About 400 g of mixed feces was collected, and 10% tartaric acid was added to ¼ of the fecal weight for nitrogen fixing. The dry matter content of the diets and feces was determined by drying the samples at 60 °C for 48 h. Additionally, the samples of the dried diets and feces were ground in feedstuff mill (KRT-34, KunJie, Beijing, China) such that they could be passed through a 1 mm screen.

The DM, crude protein (CP), and ether extract (EE) concentrations in both the diets and feces were analyzed according to the methods of the Association of Official Analytical Chemics (AOAC) [18]. The neutral detergent fiber (NDF) and acid detergent fiber (ADF) were analyzed following the method described by Van Soest et al. [19]. The acid-insoluble ash (AIA) ratio technique was used to determine the apparent total tract digestibility (ATTD) of the dietary nutrients. The AIA in the diets (*Ad*, g/kg) and feces (*Af*, g/kg) was analyzed according to the method described by Keulen and Young [20]. Using the concentration of AIA in the diet (*Nd*, g/kg) and in the feces (*Nf*, g/kg), the nutrient ATTD was calculated using the following formula [21]:

ATTD (%) = [1 − (*Ad* × *Nf*)/(*Af* × *Nd*)] × 100.

#### 2.3.3. Collection and Analysis of Milk Samples

The milk yield of each cow was recorded on a daily basis from one week postpartum. Milk samples that were approximately 50 mL in volume and that comprised morning and evening milk samples mixed at a 5:5 ratio were collected every 7 days. The milk protein, fat, lactose, milk urea nitrogen (MUN), and somatic cell count (SCC) were analyzed at the Beijing Dairy Cow Center. A near-infrared reflectance spectroscopy analyzer (Seris300 CombiFOSS; Foss Electric, Hillerød, Denmark), which is a seamless integration of the MilkoScanRM (Hillerød, Denmark) and FossomaticTM (Flow Cytometry, Hillerød, Denmark), was used for the determination.

#### 2.3.4. Collection and Analysis of Blood Samples

Blood was collected from the tail vein of the cows using 10 mL blood collection heparin-coated tubes before morning feeding every 7 days. Plasma was obtained via the centrifugation of the blood samples at 3500× *g* for 15 min at 4 °C and stored at −20 °C until analysis.

Glucose (GLU), non-esterified fatty acid (NEFA), β-hydroxybutyric acid (BHBA), triglycerides (TG), and total cholesterol (TC) were measured using blood colorimetric commercial kits (Beijing Jiuqiang Biotechnology, Beijing, China). Leptin (LEP), insulin (INS), glucagon (Glu), T3, and T4 were measured using ELISA kits (Linco Research, Inc., St. Charles, MO, USA). Alanine aminotransferase (ALT), aspartate aminotransferase (AST), total bilirubin (TBIL), lactate dehydrogenase (LDH), and alkaline phosphatase (ALP) were measured using biochemical methods and were analyzed using a Hitachi 7020 automated biochemistry analyzer (Hitachi Co., Tokyo, Japan).

#### 2.3.5. Collection and Analysis of Rumen Fluid

About 100 mL rumen fluid was collected from every cow using stomach tubes before morning feeding every 7 days. The first two tubes of rumen fluid were discarded during sampling to avoid measuring the pH value of the rumen fluid due to the mixing of the saliva secreted by chewing cows during intubation. The rumen fluid was immediately filtered by four layers of gauze, and the pH value was determined with a pH meter. They were then divided into a number of 15 mL centrifuge tubes and stored at −20 °C for further analysis.

The rumen fluid samples were defrosted in the laboratory at 4 °C, and the supernatant was collected at 4000 r/min for 15 min. The NH_3_-N concentration was measured by means of the phenol–sodium hypochlorite colorimetric method [22], and the VFA concentration was measured by means of the gas chromatography method (6890N, Agilent technologies, Avondale, PA, USA) with a capillary column (HP-INNOWax 19091N-213, Agilent) [23].

### 2.4. Statistical Analysis

The DMI, EB, NEI, MY, milk composition, blood and rumen fermentation parameters were analyzed using SAS (SAS version 9.4, SAS Institute Inc., Cary, NC, USA) using the MIXED procedure for the repeated measures of data, with the treatment, time, and interaction of the treatment over time acting as fixed effects. The BW, BCS, and ATTD were subjected to one-way analysis of variance (ANOVA). Statistical differences between means were determined by Tukey’s multiple comparison test. Significance was declared at *p* < 0.05.

## 3. Results

### 3.1. Body Weight, Body Condition Score, Dry Matter Intake and Energy Balance

As shown in Table 3, the changes in the BCS and BW showed no significant differences among the three groups (*p* > 0.05). Compared to the LC and MC groups, the HC group had higher prenatal EB, NEI, and DMI (*p* < 0.05), but no significant differences were observed postpartum (*p* > 0.05). Additionally, for the DMI (corn stover), no significant differences were observed among the three groups (*p* > 0.05).

### 3.2. Milk Yield and Milk Composition

No significant differences were observed for among the postpartum milk production, 4% FCM production, milk fat rate and fat yield, milk protein rate and protein yield, milk lactose rate and lactose yield, milk urea nitrogen, and SCC in three groups (*p* > 0.05, Table 4).

### 3.3. Blood Parameters

As shown in Table 5, the plasma NEFA content in the postpartum LC treatment was significantly higher than it was in the MC group (*p* < 0.05), and the content of β-hydroxybutyric acid (BHBA) in the postpartum LC group was significantly higher than it was in the other groups (*p* < 0.05). No significant differences were observed in the GLU, TG, TC, LEP, INS, Glu, T3, and T4 in prepartum, postpartum, and calving periods (*p* > 0.05).

As shown in Table 6, compared to the other groups, the HC group had a lower plasma AST and TBIL content on the day of calving (*p* < 0.05). Postpartum, the TBIL plasma concentration in the HC group was significantly lower that it was in the other groups (*p* < 0.05). No significant differences were observed in the ALT, LDH, and ALP at each time and in each period (*p* < 0.05).

### 3.4. Rumen Fermentation Parameters

As shown in Table 7, during the whole experiment, no significant differences were observed among the ruminal pH, total volatile fatty acids (TVFA), acetate, propionate, butyrate, and ammonia–N (NH_3_-N) in the three groups (*p* > 0.05).

### 3.5. Apparent Total-Tract Digestibility

As seen in Table 8, the apparent digestibility of CP, EE, and ADF in the prenatal MC and HC groups was significantly higher than it was in the LC group (*p* < 0.05). Compared to the other groups, the HC group had higher apparent DM and NDF digestibility prepartum (*p* < 0.05). However, no significant differences were observed for the apparent digestibility of all of the nutrients in the three groups postpartum (*p* > 0.05).

## 4. Discussion

Due to the rapid growth of the fetus and the demand for energy in the early stages of lactation, the energy requirements of the perinatal cow are greatly increased, but the dry matter intake of transition dairy cows decreases sharply, resulting in NEB [6,24,25]. The average DMI of the prepartum period for the three treatments was 7.04–9.94 kg/d in this experiment, which was lower than free intake of 12.6–14.3 kg/d [14], and this could be the result of the forages and concentrates being given separately and the poor quality of the forage. Additionally, there were significant differences in the DMI among the three treatment groups in prepartum. This could be due to the differences in the amount of concentrate fed to the cows; however, the NDF of corn stover was as high as 71.39% in this experiment, and the palatability was poor, and although the cows in the LC group ate relatively more corn stover, the amount was not significantly higher than it was in other groups. One study showed that when the energy density increased in the prepartum diet, a higher DMI was observed in the cows [13], but other studies showed that the DMI of postpartum dairy cows was not significantly affected by the energy levels of the prepartum period [26], and the same results were found in this experiment. The BW and BCS of dairy cows are closely related to their DMI: the higher the dry matter intake of dairy cows, the greater the increase in BW and BCS, resulting in improved milk performance in the early lactation period [27]. Moreover, Zhang et al. showed that because of the higher energy concentration of the prenatal diet, the cows will have more BW and BCS, and this will result in an improved energy balance status during the prepartum period, but there will be more loss of BW and more BCS during the first 8 weeks of lactation [13]. The results of this experiment showed better BCS changes in the HC group cows during the prepartum and postpartum periods. BW was elevated in the HC group during the prepartum period, and this group also showed the lowest amount of weight loss in the postpartum period, which indicated that increasing the ratio of concentrate in the prenatal diet in this experiment had a positive effect on improving the BW and BCS of dairy cows.

**Figure 1:**
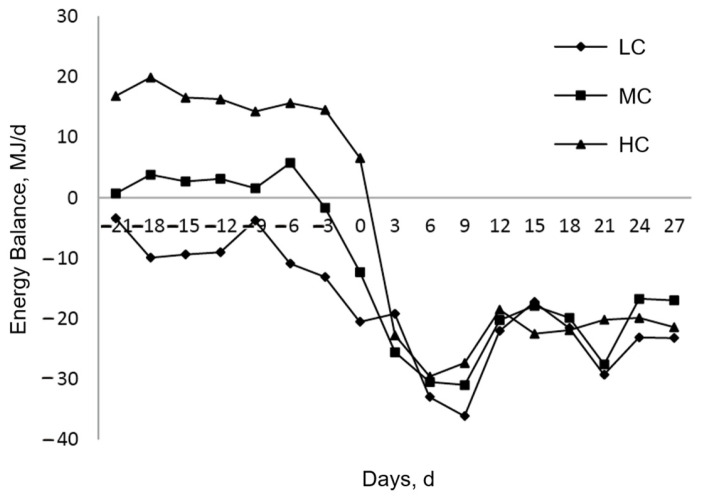
Effects of prepartum dietary concentrate levels on the energy balance of dairy cows. LC: low concentrate (0.3% of body weight), MC: medium concentrate (0.6% of body weight), HC: high concentrate (0.9% of body weight).

Increasing the proportion of dietary concentrates is accompanied by an increase in the dietary energy concentration. One research study reported that increasing the prenatal energy concentration to postpartum levels can cause the cows to adapt to the postpartum diet better, alleviating the negative energy balance in advance and having no effect on the milk performance [28]. In our research, the cows entered the NEB state regardless of the concentrate level fed to them during the prepartum period, but as the supplemental concentrate increased in the prepartum diet, the NEI increased significantly, and there was a delay before the cows entered the NEB. Additionally, the Figure 1 showed that the cows in the LC group were in the NEB at the beginning of the experiment, and the NEB continued to increase slowly as the experiment progressed. However, we found that increasing the level of concentrate in the diet resulted in a delay in the crossbred cows entering the NEB, but the onset of the NEB would be faster, which is likely to cause stress to the dairy cows. Therefore, compared to the other groups, we believe that the MC level is the most beneficial for alleviating the NEB state in dairy cows.

Milk production is closely related to DMI. As early as 1994, scholars discussed the positive correlation between milk production and DMI [29]. There are different results on the effect of feeding different energy concentration diets during the perinatal period on the milk production of postpartum dairy cows. An experiment showed that the milk production of cows with a prenatal feed intake restriction was 1.5–2.5 kg/d higher than that of free-feeding cows [12]. However, a previous study by Janovick et al. [16] showed that high-energy-fed cows had higher milk yield, and both trials showed that high-energy-fed cows had a higher milk fat rate after calving. Additionally, Janovick et al. believed that the high milk fat rate during early lactation was due to the mobilization of more body fat tissue. In our study, changing the concentrate level in the diets of Simmental crossbred cattle during the perinatal period had no significant effect on postpartum milk production, 4% FCM, and milk composition. This was likely because the cows in this experiment were Simmental crossbred cattle, which are more adaptable in terms of their energy metabolism [30]. Conversely, it could also have been because Simmental crossbred animals are not high-producing dams, and as such, the stress on these cows is less than that observed in the purebred cows that are often selected for high-level milk production.

The GLU concentration is one of the important indicators reflecting the energy metabolism level of dairy cows. It is well known that the glucose levels in the blood of dairy cows mainly come from gluconeogenesis in the liver and is caused by the propionate produced by fermentation in the rumen, which is absorbed through the rumen wall [31]. In this study, the propionate concentrations were not significantly different between the three treatment groups in each period, which may be why no significant differences were observed regarding the GLU concentration in plasma. In another study, the postpartum status of more than 80,000 dairy cows in the United Kingdom was investigated, and the BHBA and NEFA levels in the plasma were used as the criteria for judging the NEB of dairy cows after calving [32]. This may be the reason why the NEFA of the LC group was significantly higher than it was in the MC groups during the postpartum period in this experiment. However, another study found that prenatal high-energy diets could cause an NEFA increase postpartum [33], which may be the reason why there was no significant differences that were observed in the NEFA between the HC groups in this experiment. The increase in the NEFA content in the blood is the glucose-saving mechanism of dairy cows that ensures maximum lactation in conditions in which the cow is experiencing insufficient nutrition [34]. This may be the reason why the NEFA of the LC group was significantly higher than it was in the other groups during the postpartum period in this experiment. Additionally, BHBA is a sensitive NEB and fat mobilization indicator [35]. It is generally believed that when the BHBA content in the blood of a dairy cow exceeds 1.2 mmol/L, the dairy cow is considered to have subclinical ketosis [36,37,38]. In our study, the BHBA was significantly higher in the LC group than it was in the MC and HC groups, but it was far lower than 1.2 mmol/L, indicating that the cows were not at risk for ketosis in this experiment.

As important indicators of liver metabolism, AST, ALT, TBIL, and ALP can effectively reflect liver function [39]. When the liver of dairy cows is damaged, AST and ALT activity in the serum will significantly increase. TBIL is a component of the heme catabolism pathway, is important for liver function, and has been proven to reduce liver fat accumulation and high TBIL levels in the plasma that reflect liver disease [40]. In our experiment, the AST and TBIL of the cows in the LC group were significantly higher than they were in the other groups on the day of calving, and the postpartum TBIL content was still higher than it was in the other groups during postpartum, but still within a reasonable range [41]. The postpartum production performance of dairy cows was not affected by the three treatments. Therefore, under the conditions of this experiment, increasing the concentrate levels in the diets of dairy cattle will not cause damage to the livers of dairy cows.

The pH of the rumen fluid is a comprehensive indicator of the level of rumen fermentation. It is affected by many factors such as diet composition, cow saliva secretion, and rumen organic acid content [42]. Cows maintain the pH of the rumen fluid at 5.5–7.5 through a complex acid–base adjustment system [43]. In our research, the rumen fluid pH of the three groups was stable throughout the entire experimental period. This could be because the forage in this experiment was only poor-quality corn stover, which could have caused frequent rumination activity in the cows, thereby causing them to secrete more saliva to neutralize the acid in the rumen.

In the rumen, microorganisms ferment carbohydrates and other substances to produce VFAs such as acetic acid, propionic acid, butyric acid, and so on. The VFA concentration is closely related to the feed intake and diet composition of dairy cows [44]. Studies have shown that the administration of low-energy diets during the prepartum period increases the pH of the prenatal rumen fluid of dairy cows [45], and low-energy diets cause an increase in acetate production [46]. In this study, no significant differences were observed in the VFA concentration and in the ratio of acetate to propionate in the rumen, the reason for this result is likely the same as the reason explaining the pH stabilization mentioned above.

The ability of ruminants to digest feed is mainly determined by the chemical and physical properties of the feed, the activity of rumen decomposing bacteria, and the retention time of the feed in the rumen. Some studies have shown that high-energy diets show higher DMI digestibility throughout the entire digestive tract [45,47,48]. In this study, the prenatal cows in the HC group were fed the highest amount of concentrate; thus, the apparent digestibility of DM, CP, and EE in those cows was significantly higher than it was the other groups. The apparent digestibility of NDF and ADF was significantly lower than it was in the other groups of cows in the HC group during the prepartum period, which could be due to those cows having the lowest corn stover intake in the prenatal HC groups of cows and the fastest chyme flow rate.

However, by adopting the roughage first and concentrate second in the feeding methods in this experiment, we were able to observe the limiting effect that poor roughage has on the DMI of dairy cows, and this could have had have a certain impact on the results. Meanwhile, we did not include rumen microorganisms in our experiments, and further analysis of rumen microorganisms could be investigated in the future to explore the effects of different concentrate levels on the rumen microorganisms of Montbéliarde–Holstein hybrid cows.

## 5. Conclusions

This study showed that there was a significant increase in the EB, NEI, and DMI that was caused by increasing the concentrate level in prepartum diets, but no significant differences were observed in postpartum lactation performance or in milk composition. In the prepartum period, the MC diet had no negative effects on rumen fermentation and did not harm the health of the dairy cows, and it could satisfy the energy needs of dairy cows in the prepartum period. Therefore, under our experimental conditions, we suggest that a concentrate amount equaling 0.6% of body weight is the most appropriate feeding amount for prenatal Montbéliarde–Holstein crossbred cows.

## Figures and Tables

**Table 1:** Dietary composition (dry matter basis).

Items	Corn Stover	Prepartum Concentrate	Lactation TMR
Ingredient, %DM			
Chinese wildrye grass		-	20.18
Alfalfa hay		-	1.83
whole corn silage		-	15.83
Brewery mash		-	5.69
Ground corn		19.25	20.47
Barley		2.54	4.00
Wheat flour		1.75	-
Extruded soybean meal		-	7.14
Soybean meal		12.50	6.54
Rapeseed meal		-	1.47
Cottonseed meal		-	1.47
Corn germ meal		13.13	0.60
Corn husks		13.13	-
Pear residue		8.75	-
Double low rapeseed meal		7.00	-
Dry distilled grain soluble		8.75	4.00
Palm kernel meal		6.13	3.35
Molasses		3.06	0.84
Calcium carbonate		1.40	1.04
Prepartum premix ^1^		0.88	-
Lactation premix ^2^		-	2.57
Matrix Gla protein		-	0.63
Bergafat ^3^		-	0.86
Yeast culture ^4^		-	0.56
Tannic acid		-	0.09
Altamud		0.22	-
salt		-	0.33
Sodium bicarbonate		1.53	0.52
Nutrition levels, %DM			
NE_L_^5^, MJ/kg	4.31	7.41	7.14
CP	5.78	24.04	17.23
NDF	71.38	39.97	41.85
ADF	39.90	15.46	20.75
EE	2.15	2.99	5.47
Ca	0.26	0.76	0.70
P	0.24	0.83	0.36

^1^ Premix provided the following nutrition per kg (DM): vitamin A, 2,200,000 IU; vitamin D3, 550,000 IU; vitamin E, 20,000 IU; nicotinic acid, 2000 mg; Cu, 3750 mg; Mn, 5720 mg; Zn, 14,850 mg; I, 150 mg; Se, 180 mg; Co, 120 mg. ^2^ Premix provided the following nutrition per kg (DM): vitamin A, 1,000,000 IU; vitamin D3, 280,000 IU; vitamin E, 10,000 IU; nicotinic, acid 1000 mg; Cu, 3250 mg; Mn, 4800 mg; Zn, 12,850 mg; I, 140 mg; Se, 150 mg; Co, 110 mg. ^3^ Rumen fat, palm oil fatty acids (Berg + Schmidt, Hamburg, Germany); ^4^ yeast, Diamond V XP, Diamond V Mills, Inc. (Cedar Rapids, IA, USA); ^5^ net energy lactation calculated according to NRC (2001).

**Table 2:** Average nutrient ingredients ^1^.

Items ^2^	Prepartum ^3^			Postpartum
	LC	MC	HC	
Nutrition levels, %DM				
NE_L_, MJ/kg	5.32	5.76	6.04	7.14
CP	11.86	14.50	16.00	17.23
NDF	64.40	59.00	56.00	41.85
ADF	32.89	29.10	26.96	20.75
EE	3.10	3.30	3.50	5.47
Ca	0.51	0.60	0.62	0.70
P	0.41	0.50	0.55	0.36

^1^ Calculated by the final feed intake of concentrate and corn stover; ^2^ DM: dry matter, NEL: net energy for lactation, CP: crude protein, NDF: neutral detergent fiber, ADF: acid detergent fiber, EE: ether extract, NEL: NEL was a calculated value, while the others were measured values; ^3^ LC: low concentrate (0.3% of body weight), MC: medium concentrate (0.6% of body weight), HC: high concentrate (0.9% of body weight).

**Table 3:** Effects of different prenatal concentrate levels on the body weight and energy balance of dairy cows.

Items ^1^	Time ^2^	Treatment ^3^			SEM ^4^	*p* Value		
		LC	MC	HC		Treatment	Time ^5^	Interaction ^6^
BCS	Initial BCS	3.15	3.20	3.13	0.078	0.30	-	-
	−3 to −1 w changes	0	0	0.13	0.072	0.39	-	-
	1 to 4 w changes	−0.14	−0.07	0	0.076	0.30	-	-
BW, kg	Initial BW	618.82	622.36	619.15	15.745	0.84	-	-
	−3 to −1 w changes	−5.71	3.57	9.49	4.823	0.07	-	-
	1 to 4 w changes	−28.75	−20.25	−12.17	9.850	0.46	-	-
DMI, kg/d	−3 to −1 w	7.04 ^c^	8.58 ^b^	9.94 ^a^	0.196	<0.01	0.35	0.72
	1 to 4 w	15.20	16.44	16.98	0.613	0.17	<0.01	0.86
DMI (corn stover), kg/d	−3 to −1 w	5.32	5.02	4.88	0.195	0.29	0.35	0.73
NEI, MJ/d	−3 to −1 w	48.69 ^c^	60.72 ^b^	71.23 ^a^	1.711	<0.01	0.37	0.73
	1 to 4 w	109.33	116.73	122.01	3.901	0.11	<0.01	0.74
EB, MJ/d	−3 to −1 w	−9.50 ^c^	12.04 ^b^	15.48 ^a^	1.228	<0.01	0.19	0.26
	1 to 4 w	−22.04	−19.80	−18.45	5.886	0.57	0.07	0.55

^a^, ^b^, ^c^: different superscripts within a row show significant differences (*p* ≤ 0.05); ^1^ BCS: body condition score, BW: body weight, EB: energy balance, NEI: net energy intake, DMI: dry matter intake; ^2^ −3 to −1 w changes: changes from weeks 3 to 1 prepartum; 1 to 4 w: changes from weeks 1 to 4 week postpartum; −3 to −1 w: changes from weeks 3 to 1 prepartum; 1 to 4 w: changes from weeks 1 to 4 postpartum; ^3^ LC: low concentrate (0.3% of body weight), MC: medium concentrate (0.6% of body weight), HC: high concentrate (0.9% of body weight); ^4^ SEM: standard error of the mean; ^5^ sampling time effect; ^6^ interaction: the interaction between the sampling time and the treatment group.

**Table 4:** Effects of different prenatal concentrate levels on milk production and milk composition of dairy cow.

Items ^1^	Time ^3^	Treatment ^4^			SEM ^5^	*p* Value		
		LC	MC	HC		Treatment	Time ^6^	Interaction ^7^
Milk yield, kg/d	1 to 4 w	27.37	28.44	28.53	2.062	0.76	<0.01	0.96
4%FCM ^1^ yield, kg/d	1 to 4 w	27.35	28.82	28.69	2.769	0.84	<0.01	0.99
Milk fat rate, %	1 to 4 w	4.17	4.27	4.15	0.186	0.89	<0.01	0.94
Milk protein rate, %	1 to 4 w	3.36	3.44	3.41	0.087	0.77	<0.01	0.85
Milk lactose rate, %	1 to 4 w	5.03	4.98	5.08	0.065	0.33	0.06	0.67
Milk Urea Nitrogen, mg/dL	1 to 4 w	13.60	12.89	14.68	1.110	0.19	0.25	0.55
SCC ^2^, ×1000/mL	1 to 4 w	195.53	181.75	180.90	59.650	0.51	0.67	0.97
Fat yield, kg/d	1 to 4 w	1.13	1.16	1.11	0.105	0.82	<0.01	0.76
Protein yield, kg/d	1 to 4 w	0.90	0.96	0.94	0.067	0.81	<0.01	0.72
Lactose yield, kg/d	1 to 4 w	1.37	1.44	1.42	0.093	0.59	<0.01	0.89

^1^ 4%FCM: 4% fat corrected milk = (kg milk × 0.4) + (kg fat × 15); ^2^ SCC: somatic cell count; ^3^ weeks 1 to 4 in the postpartum period; ^4^ LC: low concentrate (0.3% of body weight), MC: medium concentrate (0.6% of body weight), HC: high concentrate (0.9% of body weight); ^5^ SEM: Standard error of the mean; ^6^ sampling time effect; ^7^ interaction: the interaction between the sampling time and the treatment group.

**Table 5:** Effects of different prenatal concentrate levels on the biochemical plasma indicators of prepartum and postpartum dairy cows.

Items ^1^	Time ^2^	Treatment ^3^			SEM ^4^	*p* Value		
		LC	MC	HC		Treatment	Time ^5^	Interaction ^6^
GLU, mmol/L	−3 to −1 w	3.32	3.46	3.49	0.216	0.88	0.88	0.81
	0	5.28	5.10	5.08	0.571	0.96	-	-
	1 to 4 w	3.29	3.53	3.37	0.252	0.80	0.02	0.67
TG, mmol/L	−3 to −1 w	0.39	0.38	0.42	0.044	0.82	0.36	0.99
	0	0.20	0.16	0.22	0.030	0.44	-	-
	1 to 4 w	0.165	0.20	0.19	0.022	0.30	0.10	0.23
NEFA, µmol/L	−3 to −1 w	673.81	646.69	632.03	54.975	0.93	0.42	0.91
	0	982.94	841.62	703.22	101.110	0.18	-	-
	1 to 4 w	815.56 ^a^	598.69 ^b^	759.75 ^ab^	64.107	<0.01	0.07	0.27
BHBA, mmol/L	−3 to −1 w	0.38	0.35	0.36	0.033	0.83	0.15	0.30
	0	0.42	0.47	0.35	0.077	0.55	-	-
	1 to 4 w	0.50 ^a^	0.39 ^b^	0.39 ^b^	0.040	0.05	<0.01	0.90
TC, mmol/L	−3 to −1 w	3.58	3.58	3.78	0.219	0.76	<0.01	0.27
	0	3.40	3.17	3.36	0.183	0.63	-	-
	1 to 4 w	3.94	3.77	3.97	0.218	0.78	<0.01	0.69
LEP, ng/ML	−3 to −1 w	5.84	5.88	6.06	0.232	0.78	0.05	0.21
	0	5.92	6.21	6.10	0.201	0.58	-	-
	1 to 4 w	6.17	6.27	6.53	0.202	0.46	0.20	0.45
INS, Μiu/Ml	−3 to −1 w	10.26	11.08	11.14	5.101	0.42	0.61	0.20
	0	10.04	11.92	11.49	1.530	0.68	-	-
	1 to 4 w	9.88	10.72	11.43	0.889	0.49	0.55	0.78
Glu, pg/Ml	−3 to −1 w	143.19	145.06	148.36	8.220	0.91	0.30	0.05
	0	148.66	157.40	153.21	13.469	0.91	-	-
	1 to 4 w	175.99	156.83	171.03	16.077	0.69	0.13	0.78
T3, ng/Ml	−3 to −1 w	2.11	2.03	2.14	0.147	0.87	0.07	0.32
	0	2.25	2.26	2.07	0.177	0.69	-	-
	1 to 4 w	2.32	2.41	2.28	0.088	0.55	<0.01	0.43
T4, ng/Ml	−3 to −1 w	105.00	111.22	119.80	9.235	0.54	<0.01	0.92
	0	70.39	97.59	81.80	10.946	0.24	-	-
	1 to 4 w	92.05	98.54	99.53	6.963	0.72	<0.01	0.73

^a, b^: different superscripts within a row represent significant differences (*p* ≤0.05); ^1^ GLU: glucose, TG: triglyceride, NEFA: nonesterified fatty acids, BHBA: β-hydroxybutyric acid, TC: total cholesterol, LEP: leptin, INS: insulin, Glu: glucagon, T3: triiodothyronine, T4: thyroxine; ^2^ −3 to −1 w: weeks 3 to 1 prepartum; 0: day of calving, weeks 1 to 4 postpartum; ^3^ LC: low concentrate (0.3% of body weight), MC: medium concentrate (0.6% of body weight), HC: high concentrate (0.9% of body weight); ^4^ SEM: standard error of the mean; ^5^ sampling time effect; ^6^ interaction: The interaction between the sampling time and treatment group.

**Table 6:** Effects of different prenatal concentrate levels on the liver metabolite indicators of prepartum and postpartum dairy cows.

Items ^1^	Time ^2^	Treatment ^3^			SEM ^4^	*p* Value		
		LC	MC	HC		Treatment	Time ^5^	Interaction ^6^
ALT, U/L	−3 to −1 w	21.21	23.80	22.93	1.761	0.58	0.07	0.55
	0	16.00	22.28	19.95	3.16	0.39	-	-
	1 to 4 w	20.11	22.19	21.19	1.94	0.14	0.06	0.34
AST, U/L	−3 to −1 w	52.22	52.58	55.14	6.179	0.94	0.04	0.47
	0	78.95 ^a^	66.60 ^b^	52.70 ^c^	14.065	0.04	-	-
	1 to 4 w	73.98	76.17	60.81	9.112	0.44	<0.001	0.24
TBIL, µmol/L	−3 to −1 w	1.81	1.52	1.74	0.236	0.67	0.19	0.10
	0	2.42 ^a^	1.93 ^ab^	1.62 ^b^	0.171	0.02	-	-
	1 to 4 w	2.97 ^a^	2.27 ^b^	2.06 ^c^	0.294	0.03	0.02	0.40
LDH, U/L	−3 to −1 w	418.42	446.26	417.97	23.384	0.63	0.11	0.60
	0	469.05	482.02	448.32	31.502	0.75	-	-
	1 to 4 w	596.21	629.11	522.34	47.621	0.35	0.71	0.69
ALP, U/L	−3 to −1 w	34.16	38.31	32.10	3.690	0.50	0.23	0.80
	0	34.79	43.24	34.59	4.803	0.39	-	-
	1 to 4 w	34.07	39.17	36.33	4.172	0.70	0.10	0.94

^a, b, c^: different superscripts within rows represent significant differences (*p* ≤ 0.05); ^1^ ALT: alanine aminotransferase, AST: aspartate aminotransferase, TBIL: total bilirubin, LDH: lactate dehydrogenase, ALP: alkaline phosphatase; ^2^ −3 to −1 w: weeks 3 to 1 prepartum; 0: day of calving, weeks 1 to 4 postpartum; ^3^ LC: low concentrate (0.3% of body weight), MC: medium concentrate (0.6% of body weight), HC: high concentrate (0.9% of body weight); ^4^ SEM: standard error of the mean; ^5^ sampling time effect; ^6^ interaction: the interaction between the sampling time and the treatment group.

**Table 7:** Effects of different prenatal concentrate levels on the rumen fermentation parameters of prepartum and postpartum dairy cows.

Items	Time ^3^	Treatment ^4^			SEM ^5^	*p* Value		
		LC	MC	HC		Treatment	Time ^6^	Interaction ^7^
PH	−3 to −1 w	7.18	7.13	7.09	0.050	0.31	0.22	0.18
	1 to 4 w	7.01	7.03	7.09	0.555	0.58	<0.01	0.94
TVFA ^1^, mmol/L	−3 to −1 w	58.05	60.62	62.70	4.518	0.90	0.33	0.75
	1 to 4 w	56.45	57.21	56.85	4.019	0.96	0.59	0.77
Acetate, mmol/L	−3 to −1 w	40.19	41.09	41.13	3.277	0.92	0.43	0.68
	1 to 4 w	36.99	36.78	35.77	2.57	0.94	0.26	0.44
Propionate, mmol/L	−3 to −1 w	9.64	9.99	10.24	0.780	0.80	0.10	0.59
	1 to 4 w	11.70	11.59	11.58	1.077	0.91	<0.01	0.79
Butyrate, mmol/L	−3 to −1 w	4.74	4.96	4.67	0.396	0.87	0.27	0.58
	1 to 4 w	5.68	5.36	5.56	0.638	0.94	0.72	0.71
A/P ^2^	−3 to −1 w	4.16	4.11	4.01	0.080	0.50	0.05	0.29
	1 to 4 w	3.33	3.36	3.26	0.131	0.89	<0.01	0.18
NH_3_-N, mg/dL	−3 to −1 w	5.67	5.92	5.99	0.599	0.92	0.18	0.47
	1 to 4 w	7.99	9.02	8.17	0.534	0.24	<0.01	0.97

^1^ TVFA: total volatile fatty acids. ^2^ A/P: acetate: propionate. ^3^ −3 to −1 w: weeks 3 to 1 prepartum, 0: the day of calving, weeks 1 to 4 postpartum. ^4^ LC: low concentrate (0.3% of body weight), MC: medium concentrate (0.6% of body weight), HC: high concentrate (0.9% of body weight); ^5^ SEM: standard error of the mean; ^6^ sampling time effect; ^7^ interaction: the interaction between sampling time and treatment group.

**Table 8:** Effects of different prenatal concentrate levels on the apparent nutrient digestibility of prepartum and postpartum dairy cows.

Items ^1^	Time ^2^	Treatment ^3^			SEM ^4^	*p* Value
		LC	MC	HC		
DM, %	−3 to −1 w	52.93 ^b^	54.04 ^ab^	56.83 ^a^	1.965	0.05
	1 to 4 w	73.46	69.93	72.59	2.123	0.41
CP, %	−3 to −1 w	53.97 ^b^	62.32 ^a^	63.23 ^a^	2.193	0.02
	1 to 4 w	79.24	78.40	76.37	1.931	0.57
EE, %	−3 to −1 w	65.95 ^b^	70.29 ^a^	71.23 ^a^	1.616	0.02
	1 to 4 w	84.67	82.19	81.16	2.799	0.29
NDF, %	−3 to −1 w	59.11 ^a^	57.36 ^a^	52.33 ^b^	2.24	0.03
	1 to 4 w	59.30	59.31	59.35	3.347	0.63
ADF, %	−3 to −1 w	49.61 ^a^	46.51 ^b^	45.78 ^b^	2.219	0.05
	1 to 4 w	54.16	54.35	54.73	3.535	0.52

^a, b^: different superscripts within rows represent significant differences (*p* ≤ 0.05); ^1^ DM: dry matter, CP: crude protein, EE: ether extract, NDF: neutral detergent fiber, ADF: acid detergent fiber; ^2^ −3 to −1 w: 3 to 1 week in prepartum, 1 to 4 weeks in postpartum; ^3^ LC: low concentrate (0.3% of body weight), MC: medium concentrate (0.6% of body weight), HC: high concentrate (0.9% of body weight); ^4^ SEM: standard error of the mean.

## Data Availability

Not applicable.
